# Cross-Linked Regulation of Coral-Associated Dinoflagellates and Bacteria in *Pocillopora* sp. during High-Temperature Stress and Recovery

**DOI:** 10.3390/microorganisms9091972

**Published:** 2021-09-16

**Authors:** Jiayuan Liang, Chuanqi Deng, Kefu Yu, Ruiqi Ge, Yongqian Xu, Zhenjun Qin, Biao Chen, Yinghui Wang, Hongfei Su, Xueyong Huang, Wen Huang, Guanghua Wang, Sanqiang Gong

**Affiliations:** 1 Coral Reef Research Center of China, Guangxi University, Nanning 530004, China; jyliang@gxu.edu.cn (J.L.); wyh@gxu.edu.cn (Y.W.); shf2016@gxu.edu.cn (H.S.); huangxueyong@gxu.edu.cn (X.H.); wenhuang@gxu.edu.cn (W.H.); wgh@gxu.edu.cn (G.W.); 2 Guangxi Laboratory on the Study of Coral Reefs in the South China Sea, Nanning 530004, China; 3 School of Marine Sciences, Guangxi University, Nanning 530004, China; cqdeng163@163.com (C.D.); grace112237@163.com (R.G.); 18127345798@163.com (Y.X.); qzj_gxu@163.com (Z.Q.); biaochenwork@163.com (B.C.); 4 Southern Marine Science and Engineering Guangdong Laboratory (Zhuhai), Zhuhai 519080, China; 5 Key Laboratory of Tropical Marine Bio-Resources and Ecology, South China Sea Institute of Oceanology, Chinese Academy of Sciences, Guangzhou 510301, China; gongsanqiang@scsio.ac.cn

**Keywords:** Symbiodiniaceae, photosynthetic bacteria, *F_v_*/*F_m_*, photosynthetic pigment, global warming

## Abstract

As the problem of ocean warming worsens, the environmental adaptation potential of symbiotic Symbiodiniaceae and bacteria is directly related to the future and fate of corals. This study aimed to analyse the comprehensive community dynamics and physiology of these two groups of organisms in the coral *Pocillopora* sp. through indoor simulations of heat stress (which involved manually adjusting the temperature between both 26 °C and 34 °C). Heat treatment (≥30 °C) significantly reduced the abundance of Symbiodiniaceae and bacteria by more than 70%. After the temperature was returned to 26 °C for one month, the Symbiodiniaceae density was still low, while the absolute number of bacteria quickly recovered to 55% of that of the control. At this time point, the *F_v_*/*F_m_* value rose to 91% of the pretemperature value. The content of chlorophyll b associated with Cyanobacteria increased by 50% compared with that under the control conditions. Moreover, analysis of the Symbiodiniaceae subclade composition suggested that the relative abundance of C1c.C45, C1, and C1ca increased during heat treatment, indicating that they might constitute heat-resistant subgroups. We suggest that the increase in the absolute number of bacteria during the recovery period could be an important indicator of coral holobiont recovery after heat stress. This study provides insight into the cross-linked regulation of key symbiotic microbes in the coral *Pocillopora* sp. during high-temperature stress and recovery and provides a scientific basis for exploring the mechanism underlying coral adaptation to global warming.

## 1. Introduction

With global climate warming, rising sea surface temperature (SST) is a major threat to the survival and development of coral reefs [1,2,3,4]. Many large-scale coral bleaching events are related to the abnormal rise in SST [5,6,7], with the frequency and severity of coral bleaching tending to increase [8]. The most direct impact of high SST is the large-scale expulsion of endosymbiotic dinoflagellates (family Symbiodiniaceae) by coral, resulting in a loss of pigmentation and a disruption in the coral energy supply [9,10] and ultimately leading to coral bleaching or mortality. However, some species of coral hosts are adaptable and resistant to environmental stress [11,12], primarily because their symbiotic microorganisms are somewhat plastic and can positively respond to environmental stress [13,14,15,16,17,18,19,20,21,22]. Therefore, it is necessary to gain deep insight into the physiological dynamics of Symbiodiniaceae and bacteria (key members of coral–microbe assemblages), especially with respect to their coordinating role, in the coral host response to abnormal environmental stress.

According to current research, a wide variety of Symbiodiniaceae provide more than 95% of the energy to coral holobionts through photosynthesis [23,24]. When corals are stressed by heat, the density and community structure of the symbiotic Symbiodiniaceae can change substantially [25]. For example, the absolute number of symbiotic Symbiodiniaceae changes in response to seasonal variations in SST [26]. Xu et al. evaluated the correlations between the Symbiodiniaceae density, the effective photochemical efficiency, and the seasonal variation in SST in five dominant reef coral species from the northern South China Sea (Luhuitou fringing reef). The thermal tolerance of coral is primarily and positively dependent on its Symbiodiniaceae density [26]. In addition, when corals were subjected to high-temperature stress, the Symbiodiniaceae density decreased greatly. Kemp et al. investigated the community dynamics and physiology of symbiotic Symbiodiniaceae in the coral *Orbicella faveolata* before, during, and after a natural thermal bleaching event off the coast of Puerto Morelos, Mexico [25]. The researchers found that the Symbiodiniaceae density and maximum photochemical quantum yield (*F_v_*/*F_m_*) decreased sharply during a thermally induced coral bleaching event. In addition, the community structure of Symbiodiniaceae responded positively, reflected by the increase in the relative abundance of members of heat-tolerant subclades (A3 and D1a, with higher photosynthetic efficiency). Tong et al. analysed eight potential environmental factors (temperature, salinity, NH_4_^+^, NO_3_^−^, PO_4_^3−^, NO_2_^−^, dissolved oxygen, and depth) affecting the community structure of symbiotic Symbiodiniaceae in the *Galaxea fascicularis* and *Montipora* spp. corals from three biogeographic regions at different latitudes and temperatures in the South China Sea [27]. The survey results suggested that temperature played a major role in shaping the Symbiodiniaceae community structure and coral–alga symbiosis. Thus, the Symbiodiniaceae density and community structure directly influence the adaptability and tolerance of coral hosts to changing environments.

In addition, coral-associated bacteria are among the most abundant microbial organisms within coral holobionts and play an important role in energy supply, material circulation, disease occurrence, and physiological adaptability [15,28,29,30]. On the basis of operational taxonomic units (OTUs), next-generation sequencing (NGS) analysis has revealed more than a thousand species of coral-associated bacteria [31,32]. Various coral-associated bacteria respond positively to changes in the external environment. Ritchie et al. showed that when the temperature increased, the dominant community in coral (*Acropora* spp.) mucus shifted from comprising antibiotic-producing bacteria to comprising conditionally pathogenic bacteria [33], which explained the decrease in resistance and increase in the case rate of coral hosts. Studies on associated microbial communities in the hard coral *Acropora millepora* tagged on a reef flat of Magnetic Island (Great Barrier Reef, Australia) showed that the dominant species changed to *Vibrio* spp. after a bleaching event induced by high temperature [34]. This may have been caused by the bacterial community responding to the lack of symbiotic Symbiodiniaceae and the rise in SST. In addition, many studies have shown that coral-associated bacteria play an important role in the response of coral hosts to abnormal environmental conditions [18,25,35,36,37,38,39]. In the above studies, the response characteristics of Symbiodiniaceae and associated bacteria in coral to abnormal environmental conditions were comprehensively characterised. However, more efforts are still needed to better understand complex coral holobionts because they represent mutually beneficial and dynamically balanced assemblages that include actinozoans, Symbiodiniaceae, and bacteria. The complete regulatory process and response characteristics of key members to abnormal environmental stress are still unclear, especially in terms of absolute quantity and community composition.

**Figure 1:**
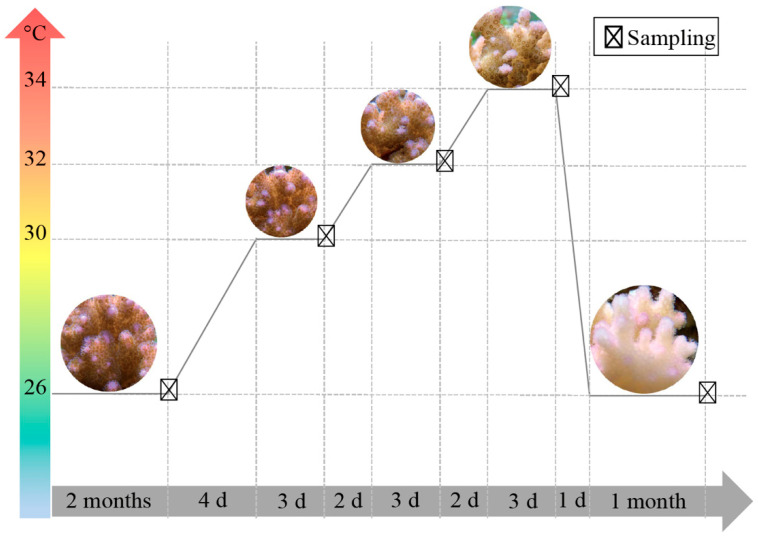
Heat treatment and sample collection. The horizontal line represents the time of the experiment. The symbol ☒ represents the sampling point at different temperatures (polar coordinates).

In the present study, we aimed to elucidate the response characteristics and coordinating roles of key symbiotic members (Symbiodiniaceae and bacteria) in the environmentally sensitive coral *Pocillopora* sp. to heat stress and early recovery. A laboratory-based simulation experiment was designed to apply different temperature stresses (Figure 1). Various comprehensive physiological parameters (community composition, photochemical efficiency, photosynthetic pigment content, and absolute cell numbers) related to Symbiodiniaceae and bacteria were analysed in detail under various temperature gradients. This study provides insights into the dynamic regulatory process of key coral symbionts (Symbiodiniaceae and bacteria) to high-temperature stress and new considerations for the adaptation of coral hosts to global warming.

## 2. Materials and Methods

### 2.1. Coral Sample Collection and Recovery in the Laboratory

*Pocillopora* sp. colonies were collected using a hammer and chisel on a dive with self-contained underwater breathing apparatus (SCUBA) gear to a depth of 4–5 m in July 2018 at the Luhuitou fringing reef (109°29′16″ E, 18°13′18″ N), Sanya city, Hainan Province, China. Six *Pocillopora* sp. colonies (approximately 20 cm in diameter) were collected, each separated by a distance greater than 10 m, and were immediately transported back to the laboratory for indoor coral cultivation. The coral species were identified mainly according to their phenotype and type of calcium carbonate skeleton. Each coral colony was subsequently cut into 5–6 nubbins (approximately 5 cm in diameter) on average and mixed in two aquarium tanks (control tank: *n* = 6, treatment tank: *n* = 27) to recover and acclimate to the new conditions for 2 months. Aquarium light (a 250 W metal halogen lamp and 4 T5HO lamps, Odyssea) was used, as its light spectrum was similar to that of natural light (12 h light:12 h dark photoperiod). The specifications and environmental parameters of the aquarium were as follows: size, 60 × 60 × 60 cm^3^ (length × width × height); carbonate hardness, 7.0 ± 0.3; environmental temperature, 26 ± 0.5 °C; salinity, 35 ± 1.5‰; pH, 8.2 ± 0.1; Ca^2+^ concentration, 400 ± 25 ppm; and Mg^2+^ concentration, 1360 ± 40 ppm. The above parameters were essentially consistent with those measured at the sampling sites. The water in the aquarium was formulated by dissolving natural sea salts in reverse-osmosis (RO) pure water and was changed weekly (1/10 volume). The temperature of water in the aquarium was measured by a thermometer and temperature sensor. The water flow rate was 6500 L/h.

### 2.2. Heat Treatment of Samples

After two months of acclimation, heat was applied by a heating device (Water Cooler, Hailea, Model: HS-66A, temperature detector: Caperplus C1) at a rate of 1 °C/day, and the overall temperature programme was as follows: 26 °C (control), 30 °C for 3 days, 32 °C for 3 days, 34 °C for 3 days, and 26 °C recovery for 1 month (Figure 1). Five coral nubbins (*n* = 5) were randomly collected within an hour before each temperature change for Symbiodiniaceae density detection, metagenomic DNA extraction, and photosynthetic pigment determination. The collected samples were not used in subsequent temperature gradients. It should be emphasised that a control tank was used as a control experiment to monitor the water quality and the stability of coral holobionts throughout the experiment (Supplementary Material Figures S2–S4 for relevant data).

### 2.3. Determination of Maximum Photochemical Efficiency

*F_v_*/*F_m_* was measured using a pulse amplitude-modulated (PAM) fluorometer (Diving-PAM, Walz, Germany) within two hours before each temperature change. The parameters were as follows: a 30 min dark adaptation period, 100 μmol/m^2^/s photosynthetically active radiation (PAR), and a 30 s time interval. PAM detection was performed underwater, and the *F_v_*/*F_m_* readings were carried out at a distance of 0.5 cm from the surface of the coral nubbins. Duplicate data of eight *F_v_*/*F_m_* were obtained from different surfaces of each coral nubbin.

### 2.4. Photosynthetic Pigment Analysis

Coral tissue (including Symbiodiniaceae and other microbial organisms) was removed from the calcium carbonate skeleton using a Waterpik^TM^ (3–5 kgf/cm^2^) containing precooled filtered seawater (0.22 μm) until only the white coral skeleton remained (Supplementary Material Figure S1). Two 100 mg portions of tissue (wet weight) were then collected from the solution obtained above via centrifugation at 13,000× *g* for 5 min. Five millilitres of 90% acetone solution was subsequently added to the collected tissue precipitate, which was then ground using an automatic rapid sample grinding instrument (Shanghai Jingxin Industrial Development Co., Ltd., Shanghai, China) for 30 s at a frequency of 50 Hz. Afterwards, the tissue debris was removed through centrifugation at 13,000× *g* for 20 min, and then the supernatant was fixed in 50 mL of 90% acetone to obtain crude pigment extracts. Five millilitres of crude pigment extract was analysed to estimate chlorophyll contents according to the methods of Jeffrey and Humphrey [40]. The following formulas were used (mg/L):

(1) $$\mathrm{Chl}\;a=11.93A_{664}-1.93A_{647}$$

(2) $$\mathrm{Chl}\;b=20.36A_{647}-5.5A_{664}$$

(3) $$\mathrm{Chl}\;c={\mathrm{Chl}\;c}_{1}+{\mathrm{Chl}\;c}_{2}=24.36A_{630}-3.73A_{664}$$

Based on the above results, the wet weight of chlorophyll (mg/g fresh weight (FW)) was equal to chlorophyll (mg/L) × 0.05 L/0.1 g. The remaining 45 mL of crude pigment extract was used to analyse carotenoid contents. The organic solvent (acetone) of the crude pigment extract was removed via N_2_ evaporation, while the chlorophyll and lipids were removed through saponification. The reaction liquid was then extracted three times with ether to obtain pure carotenoids. The carotenoid content was subsequently calculated according to the following formula [41]:

(4) $$\mathrm{Carotenoids}\;\left(\mathrm{mg}/g\;\mathrm{FW}\right)=\frac{(A_{450}\times V\times 10)/2500}{0.1}$$

where V is the total volume of ether extract, 2500 is the average extinction coefficient of carotenoids, and 0.1 is the wet tissue weight (g FW).

### 2.5. Genomic DNA Extraction and Symbiodiniaceae Subclade Analysis

We cut approximately 3 cm long short branch from each coral nubbin, removing the water attached to the surface with absorbent paper, and then added liquid nitrogen to grind the short branch into powder with a mortar. Accurately measure 90 mg of powder, which included both tissue and skeleton, was extracted for genomic DNA using a TIANamp Marine Animals DNA Kit (Tiangen Biotech (Beijing) Co., Ltd., Beijing, China) according to the manufacturer′s instructions. Before the sample was accurately weighed, water attached to the surface was removed with absorbent paper. As a DNA barcode marker for Symbiodiniaceae, the nuclear ribosomal internal transcribed spacer (ITS2) region was amplified using the specific PCR primers ITSintfor2 (5′-GAATTGCAGAACTCCGTG-3′) [42] and ITS2-reverse (5′-GGGATCCATATGCTTAAGTTCAGCGGGT-3′) [43]. PCR analysis was carried out according to the methods of Chen et al. [44]. The purified PCR amplicons were subsequently combined in equimolar amounts via a 2 × 300-bp paired-end (PE) sequencing strategy on the Illumina MiSeq platform at Majorbio Biopharm Technology Co., Ltd. (Shanghai, China). Quality control of the Illumina MiSeq Platform output data (i.e., data quality control and removal of low-quality bases at the end of the sequence and of redundant bases during sequencing) was conducted according to the methods of Bolger et al. using Trimmomatic software [45]. Fragments of ITS2 rDNA were obtained using the PEAR method developed by Zhang et al. [46]. Read quality trimming and chimaera checks were performed with MOTHUR (version 1.30.2, https://www.mothur.org/wiki/Download_mothur, accessed on 20 December 2019). Cutadapt was used to trim the reverse and forward primer sequences. Sequence alignment analysis was carried out via local BLASTN searches based on information within a nonduplicate ITS2 database established by Chen et al. [44]. The relative abundance of sequences corresponding to the Symbiodiniaceae subclade (equivalent to species) in each sample was calculated via Microsoft Excel, and the dominant subclade types were selected.

### 2.6. Determination of Symbiodiniaceae Density

Five coral nubbins were randomly extracted under each temperature gradient, and 2 cm of each nubbin was used to determine the Symbiodiniaceae density. The methods of experimental operations, including coral tissue collection, Symbiodiniaceae cell count, and coral surface area calculation, were the same as those in previous reports [26,47]. Three replicates of Symbiodiniaceae density data were obtained from each of the coral nubbins.

### 2.7. Community Composition Analysis and qPCR-Based Quantification of Associated Bacteria

The V3–V4 hypervariable region of the bacterial 16S rRNA gene was PCR amplified with 338F (5′-ACTCCTACGGGAGGCAGCAG-3′) and 806R (5′-GGACTACHVGGGTWTCTAAT-3′), for which sequence-specific barcode can be marked on each sample [48,49]. PCR analysis was carried out according to the methods of Liang et al. [32]. The purification and sequencing strategy of PCR products were the same as that in previous reports [45,50]. According to their overlap, the PE reads were merged into a sequence allowing a maximum mismatch ratio of 0.2 in the overlapping region. The merged sequences were clustered into OTUs with a 97% similarity cut-off using UPARSE software (version 7.1; http://drive5.com/uparse/, accessed on 25 December 2019). The obtained effective reads were compared with the content of the Ribosomal Database Project (RDP; http://rdp.cme.msu.edu/, accessed on 25 December 2019), and then the RDP classifier (version 2.11; https://sourceforge.net/projects/rdp-classifier/, accessed on 25 December 2019) was used to annotate the sequences to obtain species information according to the description in our previous paper [32]. When the taxonomic information of each OTU was obtained, the bacterial community composition of each sample was determined at each taxonomic level (family, genus, species, etc.). The taxonomy was aligned and compared with the content of the SILVA database (release 123; http://www.arb-silva.de, accessed on 25 December 2019) (Quast et al., 2013) using the QIIME platform (version 1.9.1; http://qiime.org/scripts/assign_taxonomy.html, accessed on 25 December 2019). Based on OTU cluster analysis, the community richness (ACE) and community diversity (Shannon) indices were estimated for each sample using the MOTHUR software (version 1.30.2) [51].

Quantitative PCR in conjunction with SYBR Green I was performed on a specific 172-bp region of the bacterial 16S rRNA gene via Eub338 (5′-ACTCCTACGGGAGGCAGCAG-3′) and Eub518 (5′-ATTACCGCGGCTGCTGG-3′) primers to measure the absolute number of bacterial cells in a 90 mg coral sample. According to the corresponding relationship between mass and surface area of coral *Pocillopora* sp., the surface area corresponding to the 90 mg sample can be accurately calculated, and then the number of bacterial cells in coral unit surface area (cm^−2^) can be calculated (Supplementary Material Figure S5). The qPCR was performed using a 9600 Plus fluorescence quantitative PCR instrument (Bioer, Hangzhou, China). The 20 μL reaction consisted of 10 μL of ChamQ SYBR Colour qPCR Master Mix (2×), 2 μL of extracted DNA, 5 μmol of each primer (0.4 μL), and 7.2 μL of ddH_2_O. The following cycling conditions were used: 95 °C for 3 min, followed by 35 cycles of 95 °C for 30 s, 56 °C for 30 s and 72 °C for 40 s. A melting curve was then generated. A pMD18-T plasmid with a known copy number (1.6672 × 10^10^ copies/μL) containing the region of the 16S rRNA gene PCR amplicon was serially diluted to 10^−3^–10^−8^ and then used to generate a standard curve. Strict negative controls, including buffer and water, were included in all PCR samples. Due to the linear relationship between the cycle threshold (Ct) value and the logarithm of the initial copy number of each template, the initial copy number could be calculated from the standard curve as long as the Ct value of the coral sample was obtained.

### 2.8. Statistical Analyses

The data obtained in this study included photosynthetic efficiency, photosynthetic pigment content, Symbiodiniaceae density, and absolute bacterial number. All the data were subjected to nonparametric Kruskal–Wallis tests to verify the significance of each component. The test of SNK was used for multiple comparisons. All significance levels were set to 0.05.

### 2.9. Ethical Approval and Consent to Participate

Permits for coral sampling were provided by the State Oceanic Administration, the People’s Republic of China, and the local Department of Ocean and Fisheries.

## 3. Results

### 3.1. Surface Morphology of Corals under Heat Treatment

**Figure 2:**
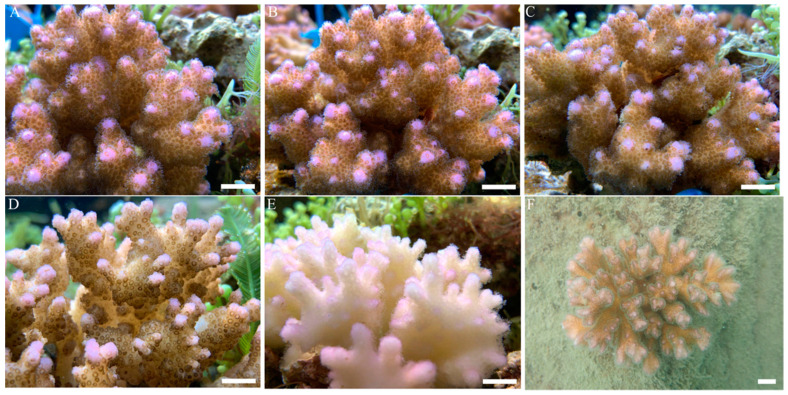
Morphological changes on the surface of one coral nubbin representative in response to increased temperature stress in an aquarium. The temperatures from (**A**–**E**) are 26 °C (control), 30 °C, 32 °C, 34 °C, and 26 °C (recovery), respectively; (**F**) shows the morphology of coral in a natural sea. (Scale bar, 1 cm).

After two months of adaptation, coral nubbins grew well, with freely stretched tentacles and bright colours, and visible growth points could be seen in the aquarium tank (Figure 2A). When the temperature of the surrounding water was raised to 30 °C for 3 days, the surface morphology and colour of the coral nubbins did not change abnormally (Figure 2B). However, when the temperature was increased from 30 to 32 °C and maintained for 3 days, the bright colours of the coral nubbins began to fade, and the tentacles were not as active as they were before. When the temperature was raised to 34 °C for 3 days, the colour of the coral nubbins became lighter, and the tentacles were completely contracted (Figure 2D). At this time, the coral nubbins endured the stress of the high-temperature threshold, and their tentacles were in a state of tight contraction. When the temperature was returned to 26 °C and for one month, the coral tentacles began to slowly resume their free extension, but the vibrant colours did not return, with the corals instead remaining in a near-white state (Figure 2E).

### 3.2. Photochemical Efficiency of Coral Nubbins under Heat Treatment

**Figure 3:**
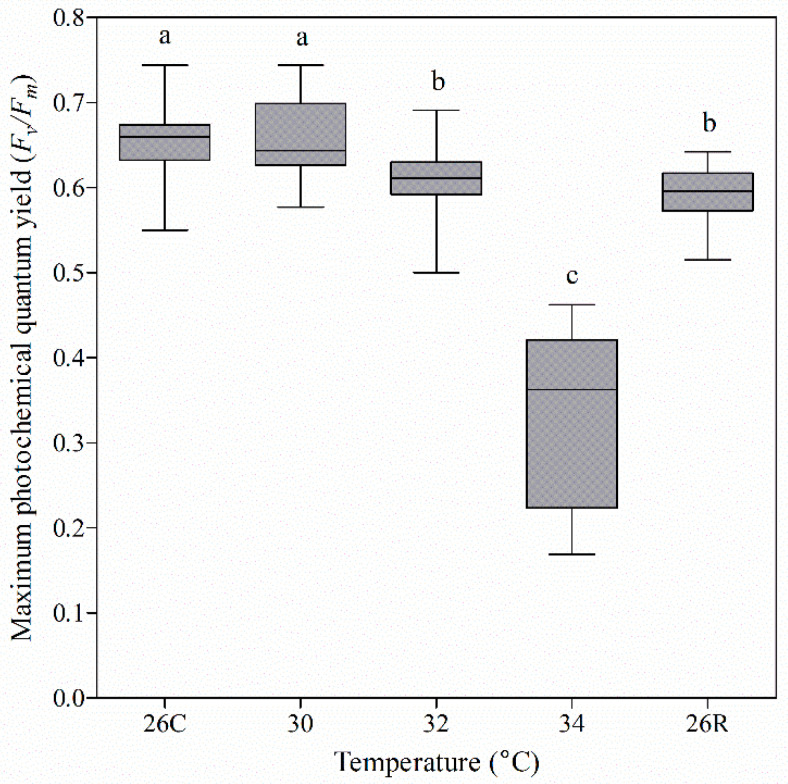
Determination of the maximum photochemical quantum yield (*F_v_*/*F_m_*) of *Pocillopora* sp. at different temperatures (means ± SEs). The coordinates 26C and 26R represent the 26 °C control and 26 °C recovery, respectively. Five biological samples (*n* = 5) were collected at the last moment of each temperature gradient for *F_v_*/*F_m_* determination. Each sample was randomly measured eight times. The data were subjected to nonparametric Kruskal–Wallis tests (same letters: no significant difference; different letters: significant difference, *p* < 0.05).

The energy source of shallow-sea scleractinian coral is provided mainly by its symbiotic organisms through photosynthesis. In this study, the photosynthetic efficiency of coral was indirectly determined from *F_v_*/*F_m_* values (Figure 3). The *F_v_*/*F_m_* values changed less in the early stage of elevated temperature stress (≤32 °C), compared with other stages. In particular, at 32 °C, the mean *F_v_*/*F_m_* value decreased slightly (by 7.57%), compared to that during the adaptation period. However, when the temperature rose to 34 °C, the coral reacted to heat stress: the average *F_v_*/*F_m_* value rapidly decreased to 50%. When the temperature was returned to 26 °C for one month, the mean *F_v_*/*F_m_* value rose to 91% of the pretemperature elevation value. In other words, the photosynthetic efficiency of coral was sensitive to the extremely high temperature (34 °C).

### 3.3. Photosynthetic Pigment Contents

**Figure 4:**
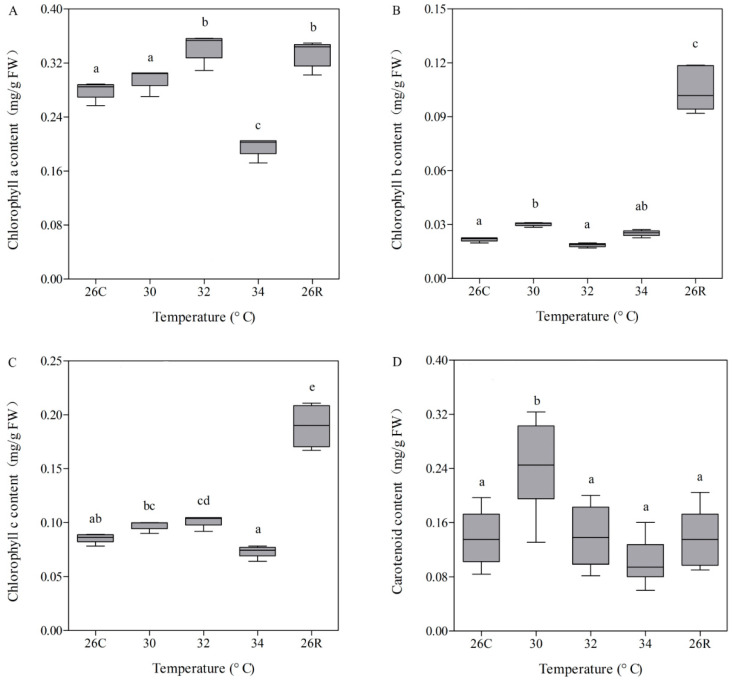
Contents of photosynthetic pigments, including chlorophyll a, b, and c (**A**–**C**, respectively) and carotenoids (**D**), in *Pocillopora* sp. tissue at different temperatures (means ± SEs). The coordinates 26C and 26R represent the 26 °C control and 26 °C recovery, respectively. Five biological samples (*n* = 5) were collected at the last moment of each temperature gradient for photosynthetic pigment determination. The data were subjected to nonparametric Kruskal–Wallis tests (same letters: no significant difference; different letters: significant difference, *p* < 0.05).

Photosynthetic pigments participate in the absorption and transfer of light energy, making the primary photochemical reactions in photosynthesis indispensable to the coral energy supply. To assess the contents of the photosynthetic pigments in coral tissues under different conditions, chlorophyll (chlorophyll a, b, and c) and carotenoids were extracted. The chlorophyll a, b, and c contents were less negatively affected from 30 to 34 °C, with the exception that chlorophyll a decreased rapidly at 34 °C (Figure 4). However, when the temperature was restored to 26 °C, the chlorophyll b and c contents rebounded rapidly, presenting a mean value that was 53% higher than the mean value before heating (Figure 4B,C). Moreover, compared with the chlorophyll contents, the carotenoid content was less sensitive to heat stress and recovery, except when the mean carotenoid content increased by 71% at 30 °C relative to the preheat mean value (Figure 4D). Overall, the change in photosynthetic pigment content was similar to that of *F_v_*/*F_m_* throughout the temperature stress cycle.

### 3.4. Symbiodiniaceae Density and Subclade Composition

**Figure 5:**
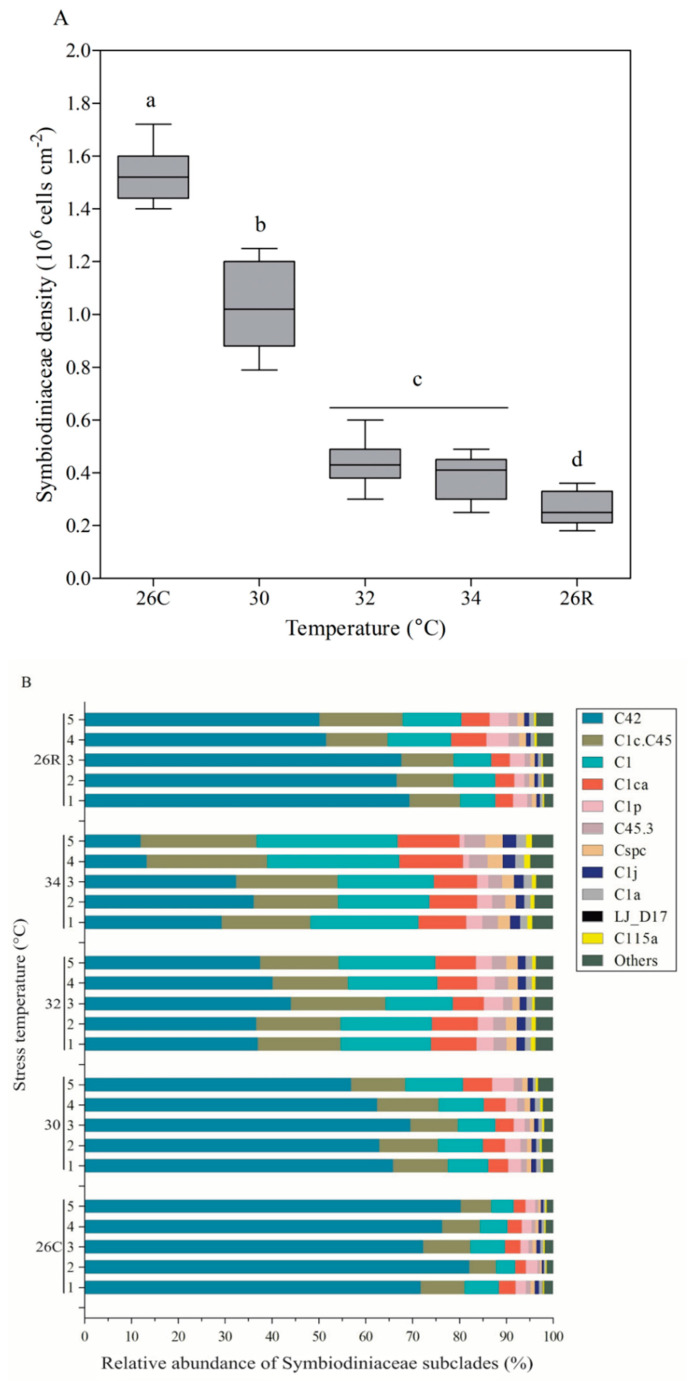
Change in Symbiodiniaceae density (**A**) and ITS2 sequence-based subclade composition (**B**) of *Pocillopora* sp. at different temperatures. The data in (**A**) are the means ± SEs. “Others” (**B**) represent Symbiodiniaceae subclades whose members have a relative abundance of less than 0.01%. The coordinates 26C and 26R represent the 26 °C control and 26 °C recovery, respectively. Five biological samples (*n* = 5) were collected at the last moment of each temperature gradient to measure Symbiodiniaceae density and extract metagenomic DNA. Three replicates of Symbiodiniaceae density data were obtained for each sample. The data were subjected to nonparametric Kruskal–Wallis tests (same letters: no significant difference; different letters: significant difference, *p* < 0.05).

Resident photosynthetic Symbiodiniaceae in coral hosts are critical to the energy supply of the host and are particularly sensitive to heat stress (Figure 5). Under normal conditions (26 °C), the mean Symbiodiniaceae density in *Pocillopora* sp. was highest, at 1.53 ± 0.03 × 10^6^ cells/cm. When coral nubbins were subjected to heat stress (varying from 30 to 34 °C), the Symbiodiniaceae density decreased rapidly to 0.39 ± 0.02 × 10^6^ cells/cm. However, when the temperature returned to 26 °C and for one month, the Symbiodiniaceae density in *Pocillopora* sp. did not recover to its original level but remained at a rather low level (0.27 ± 0.02 × 10^6^ cells/cm). Moreover, the relative abundance of dominant Symbiodiniaceae subclades significantly changed with the decrease in Symbiodiniaceae density under heat stress (Figure 5B). These dominant subclades included C42, C1c.C45, C1, and C1ca, constituting more than 90% of the total relative abundance within *Pocillopora* sp. before heat stress; all of these subclades are members of the Symbiodiniaceae clade C (equivalent to genera). With increasing temperature, there was little change in the total relative abundance of the four dominant Symbiodiniaceae subclades, while the relative abundance of each individual subclade varied greatly (Figure 5B). The relative abundance of the most dominant subclade (C42) decreased from a mean value of 76 ± 1% to 25 ± 5% with increasing temperature (from 30 to 34 °C). The relative abundance of the other three dominant subclades (C1c.C45, C1, and C1ca) also increased. When the temperature was restored to 26 °C, the relative abundance of all the dominant subclades essentially returned to preheating levels. In addition, the relative abundance of nondominant Symbiodiniaceae subclades did not change significantly during the heating cycle. According to these results, C42 is a sensitive Symbiodiniaceae subclade, and C1c.C45, C1, and C1ca are subclades whose members are tolerant to heat stress.

### 3.5. Community Composition and Absolute Number of Photosynthetic Bacteria

**Figure 6:**
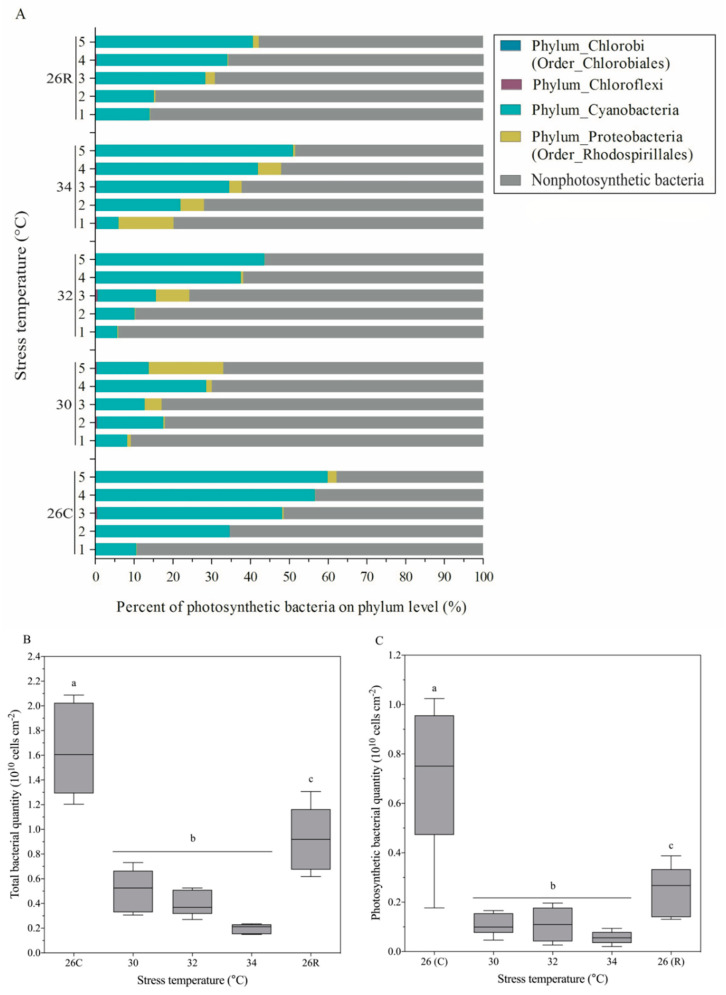
Changes in bacterial community composition and absolute numbers in the coral *Pocillopora* sp. at different temperatures: (**A**) shows the relative abundance of photosynthetic bacteria at the phylum level; (**B**,**C**) show the absolute numbers of total bacteria and photosynthetic bacteria, respectively, in 90 mg of coral. The data in (**B**,**C**) are the means ± SEs. The coordinates 26C and 26R represent the 26 °C control and 26 °C recovery, respectively. Five biological samples (*n* = 5) were collected at the last moment of each temperature gradient for bacterial composition and quantification determination. Six replicates of the bacterial quantification data were obtained for each sample. The data were subjected to nonparametric Kruskal–Wallis tests (same letters: no significant difference; different letters: significant difference, *p* < 0.05).

Sequence analysis indicated that the photosynthetic bacteria associated with *Pocillopora* sp. were mainly composed of one bacterial phylum (Cyanobacteria) and one bacterial order (Rhodospirillales) (Figure 6A). The predominant photosynthetic bacteria were Cyanobacteria, with a 42 ± 8% mean relative abundance in *Pocillopora* sp. at 26 °C. In the heat-treated samples, the relative abundance of Rhodospirillales increased slightly. Overall, the relative abundance of photosynthetic bacteria decreased, with mean values of 21 ± 4% and 24 ± 7% at 30 °C and 32 °C, respectively. However, when the temperature increased to 34 °C, the relative abundance of photosynthetic bacteria increased, with a mean value of 37 ± 6%. When the temperature was restored to 26 °C, this relative abundance decreased slightly. Analysis of the absolute number of photosynthetic bacteria showed that these coral-associated bacteria reacted rapidly to heat stress depending on their abundance (Figure 6C). With increasing temperature, the absolute number of photosynthetic bacteria decreased rapidly from a mean value of 7.01 ± 0.35 × 10^9^ cells/cm^2^ coral surface area under normal 26 °C conditions to 0.56 ± 0.05 × 10^9^ bacterial cells/cm^2^ under 34 °C conditions. When the temperature returned to and was maintained at 26 °C, the absolute number of photosynthetic bacteria rebounded substantially—to 2.52 ± 0.12 × 10^9^ cells/cm^2^. The variation in the total bacterial content was similar to that in the absolute number of photosynthetic bacteria throughout the heat-stress cycle (Figure 6B).

## 4. Discussion

The community dynamics and physiology of symbiotic Symbiodiniaceae and bacteria have been extensively studied in terms of the response of coral to heat stress. The members of unique Symbiodiniaceae subclades (e.g., D1a and A3) are known to be related to the thermal tolerance of coral [25]. However, the relative abundance of the members of the three dominant subclades, i.e., C1c.C45, C1, and C1ca, in the coral *Pocillopora* sp. increased significantly under high-temperature stress in this study. This phenomenon may be directly related to the maintenance of the energy supply when coral is in crisis, but further experimental verification is needed. Symbiotic Symbiodiniaceae played a positive regulatory role when *Pocillopora* sp. was exposed to high-temperature stress. In addition, some pathogenic bacteria thrive in high-temperature environments and become highly abundant, causing pathological changes in and bleaching of their coral hosts [33]. In fact, symbiotic Symbiodiniaceae and bacteria in coral have been separately investigated in many studies [20,21,25,26,27,28,31]. We know that coral is a typical mutually beneficial symbiotic organism (the coral holobiont) that can be actively and dynamically regulated in response to environmental change. Therefore, a comprehensive understanding of the microecological regulation of the coral holobiont (including Symbiodiniaceae and bacteria) in response to temperature is helpful for a more in-depth understanding of the mechanism and evolution of coral responses to global warming. In this study, the community dynamics and physiology of Symbiodiniaceae and bacteria (including the *F_v_*/*F_m_*, Symbiodiniaceae density and subclade composition, bacterial community composition, and absolute number, and photosynthetic pigment content) during the coral response to heat stress were systematically analysed. We have generated several novel conclusions, which are discussed below.

### 4.1. Changes in Photosynthetic Pigments and Fv/Fm Were Inconsistent with Changes in Symbiodiniaceae Density and Subclade Composition

After the planula stage, coral begins to settle on the calcareous remains of their ancestors. Most of its energy originates from photosynthesis because coral is a typical mutually beneficial holobiont that has a large number of partners (e.g., Symbiodiniaceae and photosynthetic bacteria) that provide pigments (e.g., chlorophylls and carotenoids) and carry out photosynthesis [23]. When coral faces extreme environmental stress, its energy source and maintenance can directly influence its survival. *F_v_*/*F_m_* can reflect the conversion efficiency of light energy in the photosystem II (PSII) reaction centre and is a reflection of the potential maximum photosynthetic capacity of plant and coral holobionts [26,52,53,54]. We simulated the stress of acute warming on the coral *Pocillopora* sp. to assess the influence of heat on the physiology of the coral holobiont. The content of photosynthetic pigment (chlorophyll a) and *F_v_*/*F_m_* in the coral holobiont exhibited similar change trends during the whole response to high-temperature stress and recovery. However, only the chlorophyll a and c contents increased significantly with the initial increase in temperature (at 32 °C). This phenomenon was consistent with the results reported by Nunez-Pons et al., suggesting that these contents increased presumably to meet increased metabolic demands [17]. During the recovery period, the chlorophyll (b and c) contents were also abnormally high, which may be related to the high energy demand and low member density of coral symbionts (Symbiodiniaceae and Cyanobacteria) (Figure 4). However, the content of chlorophyll b related to Cyanobacteria did not change significantly throughout the whole heat-stress process but increased significantly during the recovery period (Figure 4B). The reason behind the change of photosynthetic pigments in coral holobiont during high-temperature stress and recovery needs to be further explored. Our results are partially inconsistent with those of a previous study [17]. In a gradual thermal stress (GTS) experiment with *Exaiptasia anemones*, GTS increased chlorophyll contents and decreased Symbiodiniaceae proliferation. However, the chlorophyll contents decreased in the recovery period after GTS, while the rate of symbiont division increased [17]. Moreover, in *Exaiptasia* experiencing thermal stress and bleaching at temperatures greater than 30 °C, the remaining photosynthates in hospite symbionts continued to be translocated but at a significant cost to the organisms [55]. Photosynthesis by Symbiodiniaceae is the main energy source of coral holobionts [23,24]. Although the Symbiodiniaceae density was very low in this study (e.g., at 32 °C and during recovery at 26 °C), the relative values of *F_v_*/*F_m_* were high—94 and 91%, respectively. Basing on the changes of chlorophyll b, it is speculated that Cyanobacteria might play a key role in the photosynthetic energy supply in corals in response to high-temperature stress and recovery. Especially in the recovery period, the density of the main contributors (Symbiodiniaceae) to photosynthetic energy was low, while the absolute number of bacteria rebounded significantly (Figure 6). These findings further indicated that bacteria may play important roles in establishing a new state during coral recovery.

As we continued to analyse the changes in Symbiodiniaceae composition, we found that the original subclade composition of Symbiodiniaceae in *Pocillopora* sp. included 11 types: C42 and C1c.C45, C1, and C1ca were the dominant members, all of which belonged to Symbiodiniaceae clade C (equivalent to genera) (Figure 5B). With increasing temperature, the relative abundance of the members of the most dominant subclade, C42, decreased significantly, while that of the other dominant subclades, C1c.C45, C1, and C1ca, increased significantly. Selective pressures in environments whose temperature widely fluctuates might have adaptive value to symbiosis specificity [56]. C42 was a temperature-sensitive subgroup, and C1c.C45, C1, and C1ca were temperature-resistant subgroups. Thus, the *F_v_*/*F_m_* should be maintained mainly by the members of the temperature-tolerant subclades C1c.C45, C1, and C1ca. However, there was no sign of recovery of Symbiodiniaceae density when *Pocillopora* sp. was subjected to extreme high-temperature stress and then allowed to recover at 26 °C for one month. One of the most prominent phenomena was that the chlorophyll c content related to Symbiodiniaceae increased significantly to 225% of its original value (Figure 4C). Therefore, photosynthetic efficiency was related to not only Cyanobacteria survival but also Symbiodiniaceae survival. This could be explained by the results of the analysis of the structural composition and the absolute number of bacteria associated with *Pocillopora* sp., as described below.

### 4.2. Potential Regulatory Role of Coral-Associated Bacteria under Heat Stress

Coral-associated bacteria are very diverse and have important biological functions (material cycling, disease prevention, etc.). Previous studies have concentrated on the community dynamics of coral-associated bacteria [15,25,28,31,34], and the associated physiological functions have been focused on less [57,58]. In the present study, fluorescence quantitative PCR (absolute quantification) was used to analyse the absolute number of bacteria associated with *Pocillopora* sp. throughout the heat-stress cycle. During heating, the absolute number of bacteria decreased sharply, similar to the Symbiodiniaceae density, which was highly sensitive to high temperatures (Figure 6). However, the bacterial response was more intense in the initial heating stage (at 30 °C) than in the other stage, according to the change in numerical value. The only difference was that the absolute number of bacteria substantially recovered when the temperature returned to 26 °C for one month. This microcosm change may play an essential role in the establishment of a new state of the coral holobiont during/after extreme stress. In the face of climate variability, corals are considered particularly susceptible, and the mechanisms that contribute to their recovery must be understood [59]. Ziegler et al. pointed out that symbiotic microbial adaptation constitutes another possible mechanism to assist sensitive organisms in resisting environmental changes beyond the host’s own physiological acclimatisation and assisting the migration of heat-tolerant alleles [21]. After their numbers decreased in response to extremely high temperatures, Symbiodiniaceae members were restored in terms of composition but were still present at a very low abundance. We, therefore, speculate that Symbiodiniaceae meet the metabolic needs of coral holobionts by increasing the content of photosynthetic pigments in individual cells. In addition, the rapid recovery of coral-associated bacteria, especially photosynthetic bacteria (Cyanobacteria), may play a key role in the energy supply of the coral holobiont.

### 4.3. Model of the Coordinated Response of the Coral Holobiont during Heat Stress

**Figure 7:**
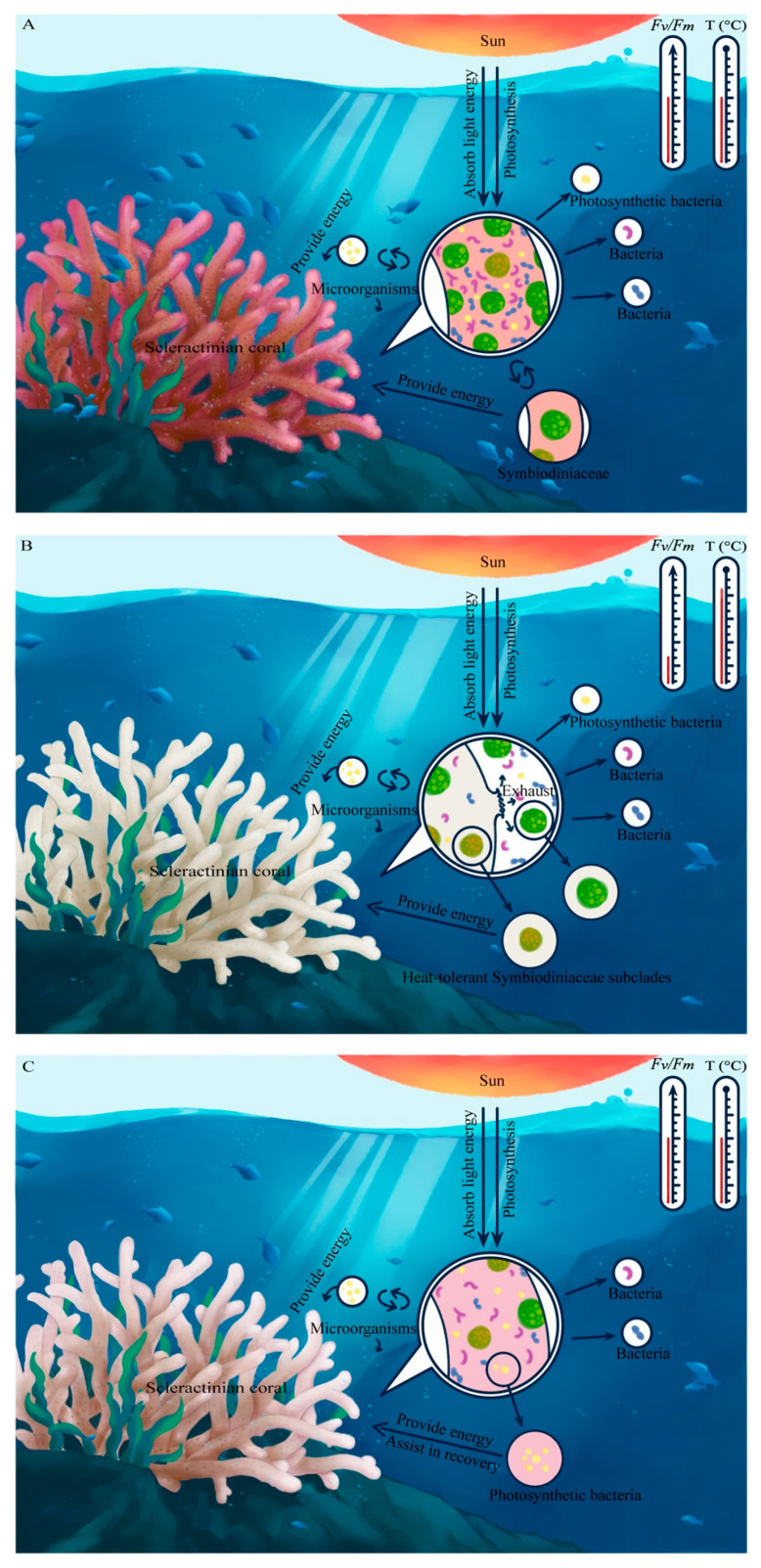
Model of the coordinated response of coral symbionts (Symbiodiniaceae and bacteria) during heat stress. The schematic illustration shows that the coral host, Symbiodiniaceae, and bacteria formed a complex holobiont with an energy supply as the core: (**A**) coral in a state of healthy growth at a suitable temperature (26 °C). At this time, coral holobionts receive enough energy from their symbiotic partners, including Symbiodiniaceae and photosynthetic bacteria, which absorb light energy for photosynthesis; (**B**) coral bleaching was significant under exposure to extremely high-temperature stress (34 °C). At this point, the most prominent feature was that large amounts of Symbiodiniaceae and bacteria were synchronously expelled from the coral hosts. The energy source of the coral holobiont was then depleted at a low *F_v_*/*F_m_*. At this point, only members of potentially heat-tolerant Symbiodiniaceae subclades provide a small amount of energy for coral holobionts; (**C**) the coral began to recover (with a normal *F_v_*/*F_m_* value) when heat stress was removed for one month. Although the Symbiodiniaceae density was very low and showed no sign of recovery, the number of bacteria (especially photosynthetic bacteria) increased to 56% of the original value; this phenomenon might play a key role in the energy supply that enabled the coral host to recover quickly. Note: photosynthetic bacteria mainly refer to cyanobacteria; illustrations in the circle indicate coral tissue.

Coral holobionts are complex and dynamic multifunctional organisms. To date, few studies have fully explored the changes in the community dynamics and physiology of symbiotic coral microbial organisms (especially the absolute quantity of bacteria) in response to environmental changes. On the basis of our laboratory simulation of high-temperature stress, a thermal response model of *Pocillopora* sp. was proposed from the surface morphology, photosynthetic pigments, symbiont composition, and Symbiodiniaceae and bacterial abundance (Figure 7). Under normal conditions, coral holobionts require energy supplied by and material circulation of symbiotic microorganisms. With respect to this point, the community structure and individual populations of symbiotic Symbiodiniaceae and bacteria in the coral host were stable, both types of organisms were highly abundant, and the coral could obtain enough energy to grow healthily (Figure 7A). However, under heat stress, the numbers of both Symbiodiniaceae and bacterial populations decreased sharply. At this time, the balance of the coral holobiont was disrupted, and by increasing the content of photosynthetic pigments per unit cell, the surviving Symbiodiniaceae and Cyanobacteria protected the basic metabolic needs of the coral holobiont. The energy supply was essentially depleted, and the coral host was at risk of mortality (Figure 7B). When the thermal stress was removed, the active bacteria that reproduce faster may play a major role in the establishment of a new state of the coral holobiont, assisting the coral host in rapid recovery (Figure 7C). Previous studies have confirmed that the members of some Symbiodiniaceae subclades (e.g., D1a and A3) are related to the heat tolerance of coral hosts [25]. This differentiation between heat-tolerant strains of Symbiodiniaceae and bacteria, including species diversity, the rate of change, and the rate of propagation, should constitute the basis for coral adaptation to environmental changes. Therefore, we speculate that coral response strategies may involve a combination of a large number of bacteria (especially highly abundant photosynthetic bacteria) with heat-tolerant Symbiodiniaceae subclade members under future warmer climates. Therefore, our study provides novel insights into the microecological regulation of key coral symbionts during heat stress and during a recovery period.

## 5. Conclusions

In this study, an indoor simulation of heat stress was conducted to elucidate the response characteristics and coordinating roles of key symbiotic members (Symbiodiniaceae and bacteria) in the environmentally sensitive coral *Pocillopora* sp. The heat stress would quickly disrupt the symbiotic relationship between coral host and microorganism (Symbiodiniaceae and bacteria). The numbers of both Symbiodiniaceae and bacterial populations decreased sharply. However, the surviving heat-tolerant Symbiodiniaceae and Cyanobacteria protected the basic metabolic needs of the coral holobiont. When the thermal stress was removed, bacteria recovered faster than Symbiodiniaceae. It meant that the active bacteria might play a major role in the establishment of a restored status of the coral holobiont, assisting the coral host in rapid recovery. Overall, we could describe a cross-linked model of two key symbiotic members (Symbiodiniaceae and bacteria) in coral *Pocillopora* sp. under heat stress and recovery. It is hypothesised that the coral response to global warming may involve a combination of a large number of bacteria with heat-resistant Symbiodiniaceae subclades.

## Data Availability

The raw data supporting the conclusions of this article will be made available by the authors, without undue reservation.

## Supplementary Materials

The following are available online at https://www.mdpi.com/article/10.3390/microorganisms9091972/s1, Figure S1: Coral skeleton removed tissue. Figure S2: Determination of the maximum photochemical quantum yield (*F_v_*/*F_m_*) of *Pocillopora* sp. in the control tank. Figure S3: Symbiodiniaceae density (A) and ITS2 sequence-based subclade composition (B) of *Pocillopora* sp. in the control tank. Figure S4: Bacterial community composition and cells density in the coral *Pocillopora* sp. in the control tank. Figure S5: Standard curve was constructed by the relationship between coral weight and surface area.

## References

[1] Hoegh-Guldberg O. (1999). Climate change, coral bleaching and the future of the world’s coral reefs. Mar. Freshw. Res..

[2] Hoegh-Guldberg O., Mumby P.J., Hooten A.J., Steneck R.S., Greenfield P., Gomez E., Harvell C.D., Sale P.F., Edwards A.J., Caldeira K. (2007). Coral reefs under rapid climate change and ocean acidification. Science.

[3] Hughes T.P., Kerry J.T., Baird A.H., Connolly S.R., Dietzel A., Eakin C.M., Heron S.F., Hoey A.S., Hoogenboom M.O., Liu G. (2018). Global warming transforms coral reef assemblages. Nature.

[4] Meissner K.J., Lippmann T., Sen Gupta A. (2012). Large-scale stress factors affecting coral reefs: Open ocean sea surface temperature and surface seawater aragonite saturation over the next 400 years. Coral Reefs.

[5] Hughes T.P., Anderson K.D., Connolly S.R., Heron S.F., Kerry J.T., Lough J.M., Baird A.H., Baum J.K., Berumen M.L., Bridge T.C. (2018). Spatial and temporal patterns of mass bleaching of corals in the Anthropocene. Science.

[6] Berkelmans R., De’ath G., Kininmonth S., Skirving W.J. (2004). A comparison of the 1998 and 2002 coral bleaching events on the Great Barrier Reef: Spatial correlation, patterns, and predictions. Coral Reefs.

[7] Edwards A.J., Clark S., Zahir H., Rajasuriya A., Naseer A., Rubens J. (2001). Coral bleaching and mortality on artificial and natural reefs in Maldives in 1998, sea surface temperature anomalies and initial recovery. Mar. Pollut. Bull..

[8] Hughes T.P., Kerry J.T., Alvarez-Noriega M., Alvarez-Romero J.G., Anderson K.D., Baird A.H., Babcock R.C., Beger M., Bellwood D.R., Berkelmans R. (2017). Global warming and recurrent mass bleaching of corals. Nature.

[9] Brown B.E. (1997). Coral bleaching: Causes and consequences. Coral Reefs.

[10] Rowan R., Knowlton N., Baker A., Jara J. (1997). Landscape ecology of algal symbionts creates variation in episodes of coral bleaching. Nature.

[11] Rowan R. (2004). Coral bleaching—Thermal adaptation in reef coral symbionts. Nature.

[12] Torda G., Donelson J.M., Aranda M., Barshis D.J., Bay L., Berumen M.L., Bourne D.G., Cantin N., Foret S., Matz M. (2017). Rapid adaptive responses to climate change in corals. Nat. Clim. Chang..

[13] Baker A.C., Starger C.J., McClanahan T.R., Glynn P.W. (2004). Corals’ adaptive response to climate change. Nature.

[14] Chen C.P., Tseng C.H., Chen C.A., Tang S.L. (2011). The dynamics of microbial partnerships in the coral Isopora palifera. ISME J..

[15] Ceh J., Raina J.B., Soo R.M., van Keulen M., Bourne D.G. (2012). Coral-Bacterial Communities before and after a Coral Mass Spawning Event on Ningaloo Reef. PLoS ONE.

[16] Howells E.J., Beltran V.H., Larsen N.W., Bay L.K., Willis B.L., van Oppen M.J.H. (2012). Coral thermal tolerance shaped by local adaptation of photosymbionts. Nat. Clim. Chang..

[17] Nunez-Pons L., Bertocci I., Baghdasarian G. (2017). Symbiont dynamics during thermal acclimation using cnidarian-dinoflagellate model holobionts. Mar. Environ. Res..

[18] Meron D., Rodolfo-Metalpa R., Cunning R., Baker A.C., Fine M., Banin E. (2012). Changes in coral microbial communities in response to a natural pH gradient. ISME J..

[19] Sampayo E.M., Ridgway T., Bongaerts P., Hoegh-Guldberg O. (2008). Bleaching susceptibility and mortality of corals are determined by fine-scale differences in symbiont type. Proc. Natl. Acad. Sci. USA.

[20] Silverstein R.N., Cunning R., Baker A.C. (2015). Change in algal symbiont communities after bleaching, not prior heat exposure, increases heat tolerance of reef corals. Glob. Chang. Biol..

[21] Ziegler M., Seneca F.O., Yum L.K., Palumbi S.R., Voolstra C.R. (2017). Bacterial community dynamics are linked to patterns of coral heat tolerance. Nat. Commun..

[22] Meron D., Maor-Landaw K., Eyal G., Elifantz H., Banin E., Loya Y., Levy O. (2020). The Complexity of the Holobiont in the Red Sea Coral *Euphyllia paradivisa* under Heat Stress. Microorganisms.

[23] Muscatine L., Mccloskey L.R., Marian R.E. (1981). Estimating the daily contribution of carbon from zooxanthellae to coral animal respiration. Limnol. Oceanogr..

[24] Falkowski P.G., Dubinsky Z., Muscatine L., Porter J.W. (1984). Light and the bioenergetics of a symbiotic coral. BioScience.

[25] Kemp D.W., Hernandez-Pech X., Iglesias-Prieto R., Fitt W.K., Schmidt G.W. (2014). Community dynamics and physiology of *Symbiodinium* spp. before, during, and after a coral bleaching event. Limnol. Oceanogr..

[26] Xu L.J., Yu K.F., Li S., Liu G.H., Tao S.C., Shi Q., Chen T.R., Zhang H.L. (2017). Interseasonal and interspecies diversities of *Symbiodinium* density and effective photochemical efficiency in five dominant reef coral species from Luhuitou fringing reef, northern South China Sea. Coral Reefs.

[27] Tong H.Y., Cai L., Zhou G.W., Yuan T., Zhang W.P., Tian R.M., Huang H., Qian P.Y. (2017). Temperature shapes coral-algal symbiosis in the South China Sea. Sci. Rep..

[28] De Castro A.P., Araujo S.D., Reis A.M.M., Moura R.L., Francini R.B., Pappas G., Rodrigues T.B., Thompson F.L., Kruger R.H. (2010). Bacterial Community Associated with Healthy and Diseased Reef Coral *Mussismilia hispida* from Eastern Brazil. Microb. Ecol..

[29] Rosenberg E., Koren O., Reshef L., Efrony R., Zilber-Rosenberg I. (2007). The role of microorganisms in coral health, disease and evolution. Nat. Rev. Microbiol..

[30] Sweet M.J., Croquer A., Bythell J.C. (2011). Bacterial assemblages differ between compartments within the coral holobiont. Coral Reefs.

[31] Li J., Chen Q., Long L.J., Dong J.D., Yang J., Zhang S. (2014). Bacterial dynamics within the mucus, tissue and skeleton of the coral Porites lutea during different seasons. Sci. Rep..

[32] Liang J.Y., Yu K.F., Wang Y.H., Huang X.Y., Huang W., Qin Z.J., Pan Z.L., Yao Q.C., Wang W.H., Wu Z.C. (2017). Distinct Bacterial Communities Associated with Massive and Branching Scleractinian Corals and Potential Linkages to Coral Susceptibility to Thermal or Cold Stress. Front. Microbiol..

[33] Ritchie K.B. (2006). Regulation of microbial populations by coral surface mucus and mucus-associated bacteria. Mar. Ecol. Prog. Ser..

[34] Bourne D., Iida Y., Uthicke S., Smith-Keune C. (2008). Changes in coral-associated microbial communities during a bleaching event. ISME J..

[35] Garren M., Azam F. (2012). Corals shed bacteria as a potential mechanism of resilience to organic matter enrichment. ISME J..

[36] Meron D., Atias E., Kruh L.I., Elifantz H., Minz D., Fine M., Banin E. (2011). The impact of reduced pH on the microbial community of the coral Acropora eurystoma. ISME J..

[37] Littman R., Willis B.L., Bourne D.G. (2011). Metagenomic analysis of the coral holobiont during a natural bleaching event on the Great Barrier Reef. Environ. Microbiol. Rep..

[38] Tout J., Siboni N., Messer L.F., Garren M., Stocker R., Webster N.S., Ralph P.J., Seymour J.R. (2015). Increased seawater temperature increases the abundance and alters the structure of natural *Vibrio* populations associated with the coral *Pocillopora damicomis*. Front. Microbiol..

[39] Thurber R.V., Willner-Hall D., Rodriguez-Mueller B., Desnues C., Edwards R.A., Angly F., Dinsdale E., Kelly L., Rohwer F. (2009). Metagenomic analysis of stressed coral holobionts. Environ. Microbiol..

[40] Jeffrey S.W., Humphrey G.F. (1975). New spectrophotometric equations for determining chlorophylls a, b, c1 and c2 in higher plants, algae and natural phytoplankton. Biochem. Physiol. Pflanz..

[41] Jensen A. (1978). Chorophylls and Carotenoids.

[42] LaJeunesse T.C., Trench R.K. (2000). Biogeography of two species of Symbiodinium (Freudenthal) inhabiting the intertidal sea anemone Anthopleura elegantissima (Brandt). Biol. Bull..

[43] Coleman A.W., Suarez A., Goff L.J. (1994). Molecular delineation of species and syngens in Volvocacean green algae (Chlorophyta). J. Phycol..

[44] Chen B., Yu K.F., Liang J.Y., Huang W., Wang G.H., Su H.F., Qin Z.J., Huang X.Y., Pan Z.L., Luo W.W. (2019). Latitudinal Variation in the Molecular Diversity and Community Composition of Symbiodiniaceae in Coral from the South China Sea. Front. Microbiol..

[45] Bolger A.M., Lohse M., Usadel B. (2014). Trimmomatic: A flexible trimmer for Illumina sequence data. Bioinformatics.

[46] Zhang J.J., Kobert K., Flouri T., Stamatakis A. (2014). PEAR: A fast and accurate Illumina Paired-End reAd mergeR. Bioinformatics.

[47] Qin Z.J., Yu K.F., Chen B., Wang Y.H., Liang J.Y., Luo W.W., Xu L.J., Huang X.Y. (2019). Diversity of Symbiodiniaceae in 15 Coral Species from the Southern South China Sea: Potential Relationship with Coral Thermal Adaptability. Front. Microbiol..

[48] Xu N., Tan G.C., Wang H.Y., Gai X.P. (2016). Effect of biochar additions to soil on nitrogen leaching, microbial biomass and bacterial community structure. Eur. J. Soil Biol..

[49] Mori H., Maruyama F., Kato H., Toyoda A., Dozono A., Ohtsubo Y., Nagata Y., Fujiyama A., Tsuda M., Kurokawa K. (2014). Design and Experimental Application of a Novel Non-Degenerate Universal Primer Set that Amplifies Prokaryotic 16S rRNA Genes with a Low Possibility to Amplify Eukaryotic rRNA Genes. DNA Res..

[50] Qin Z.J., Yu K.F., Liang J.Y., Yao Q.C., Chen B. (2020). Significant Changes in Microbial Communities Associated With Reef Corals in the Southern South China Sea During the 2015/2016 Global-Scale Coral Bleaching Event. J. Geophys. Res. Oceans.

[51] Schloss P.D., Gevers D., Westcott S.L. (2011). Reducing the Effects of PCR Amplification and Sequencing Artifacts on 16S rRNA-Based Studies. PLoS ONE.

[52] Yu X.P., Yu K.F., Huang W., Liang J.Y., Qin Z.J., Chen B., Yao Q.C., Liao Z.H. (2020). Thermal acclimation increases heat tolerance of the scleractinian coral *Acropora pruinosa*. Sci. Total Environ..

[53] Kim J.B., Lee W.C., Kim H.C., Hong S. (2020). Photosynthetic characteristics of *Pyropia yezoensis* (Ueda) Hwang & Choi measured using Diving-PAM in the Jindo-Haenam region on the southwestern coast of the Korean Peninsula. J. Appl. Phycol..

[54] Poquita-Du R.C., Goh Y.L., Huang D.W., Chou L.M., Todd P.A. (2020). Gene Expression and Photophysiological Changes in *Pocillopora acuta* Coral Holobiont Following Heat Stress and Recovery. Microorganisms.

[55] Hillyer K.E., Dias D.A., Lutz A., Roessner U., Davy S.K. (2017). Mapping carbon fate during bleaching in a model cnidarian symbiosis: The application of C-13 metabolomics. New Phytol..

[56] Rodriguez-Lanetty M., Chang S.J., Song J.I. (2003). Specificity of two temperate dinoflagellate-anthozoan associations from the north-western Pacific Ocean. Mar. Biol..

[57] Seneca F.O., Palumbi S.R. (2015). The role of transcriptome resilience in resistance of corals to bleaching. Mol. Ecol..

[58] Sogin E.M., Putnam H.M., Anderson P.E., Gates R.D. (2016). Metabolomic signatures of increases in temperature and ocean acidification from the reef-building coral, *Pocillopora damicornis*. Metabolomics.

[59] Hughes T.P., Baird A.H., Bellwood D.R., Card M., Connolly S.R., Folke C., Grosberg R., Hoegh-Guldberg O., Jackson J.B.C., Kleypas J. (2003). Climate change, human impacts, and the resilience of coral reefs. Science.

