# Supplementary material for: Cross-Linked Regulation of Coral-Associated Dinoflagellates and Bacteria in Pocillopora sp. during High-Temperature Stress and Recovery
Source: Microorganisms. 2021 Sep 16;9(9):1972. doi: 10.3390/microorganisms9091972 (PMC8468813; doi:10.3390/microorganisms9091972)
Supplement: Supplementary file 1 [file microorganisms-09-01972-s001.zip › microorganisms-1371444-supplementary.pdf]

Cross-Linked Regulation of Coral-Associated Dinoflagellates and Bacteria in *Pocillopora* sp. during High-Temperature Stress and Recovery

Jiayuan Liang[1,2,3], Chuanqi Deng[3], Kefu Yu[1,2,3,4,*], Ruiqi Ge[3], Yongqian Xu[3], Zhenjun Qin[3], Biao Chen[3], Yinghui Wang[1,2,3], Hongfei Su[1,2,3], Xueyong Huang[1,2,3], Wen Huang[1,2,3], Guanghua Wang[1,2,3] and Sanqiang Gong[5]

[1] Coral Reef Research Center of China, Guangxi University, Nanning 530004, China

[2] Guangxi Laboratory on the Study of Coral Reefs in the South China Sea, Nanning 530004, China

[3] School of Marine Sciences, Guangxi University, Nanning 530004, China

[4] Southern Marine Science and Engineering Guangdong Laboratory (Zhuhai), Zhuhai 519080, China

[5] Key Laboratory of Tropical Marine Bio-resources and Ecology, South China Sea Institute of Oceanology, Chinese Academy of Sciences, Guangzhou 510301, China

* Corresponding author, E-mail address: kefuyu@scsio.ac.cn (K. Yu), Tel & Fax: +86-771-3231358, ORCID number: 0000-0003-3409-9945

In order to monitor the stability of water quality and coral holobionts during the whole experiment, we designed a parallel aquarium tank as the control group. Coral nubbins were sampled in the early (CK1), middle (CK2) and late (CK3) stages of the whole experimental cycle to analyze the F_v/F_m (Fig. S2), Symbiodiniaceae density and subclade (Fig. S3), and the density of bacterial cells and relative abundance of photosynthetic bacteria (Fig. S4). Two to three coral nubbins were randomly selected for each CK to analyze the relevant data. It should be emphasized that all conditions of the control tank (26 °C) and the heating tank (from 26 °C to 34°C, and then back to 26 °C) remained the same except that the temperature was different.

Figures

Fig. S1

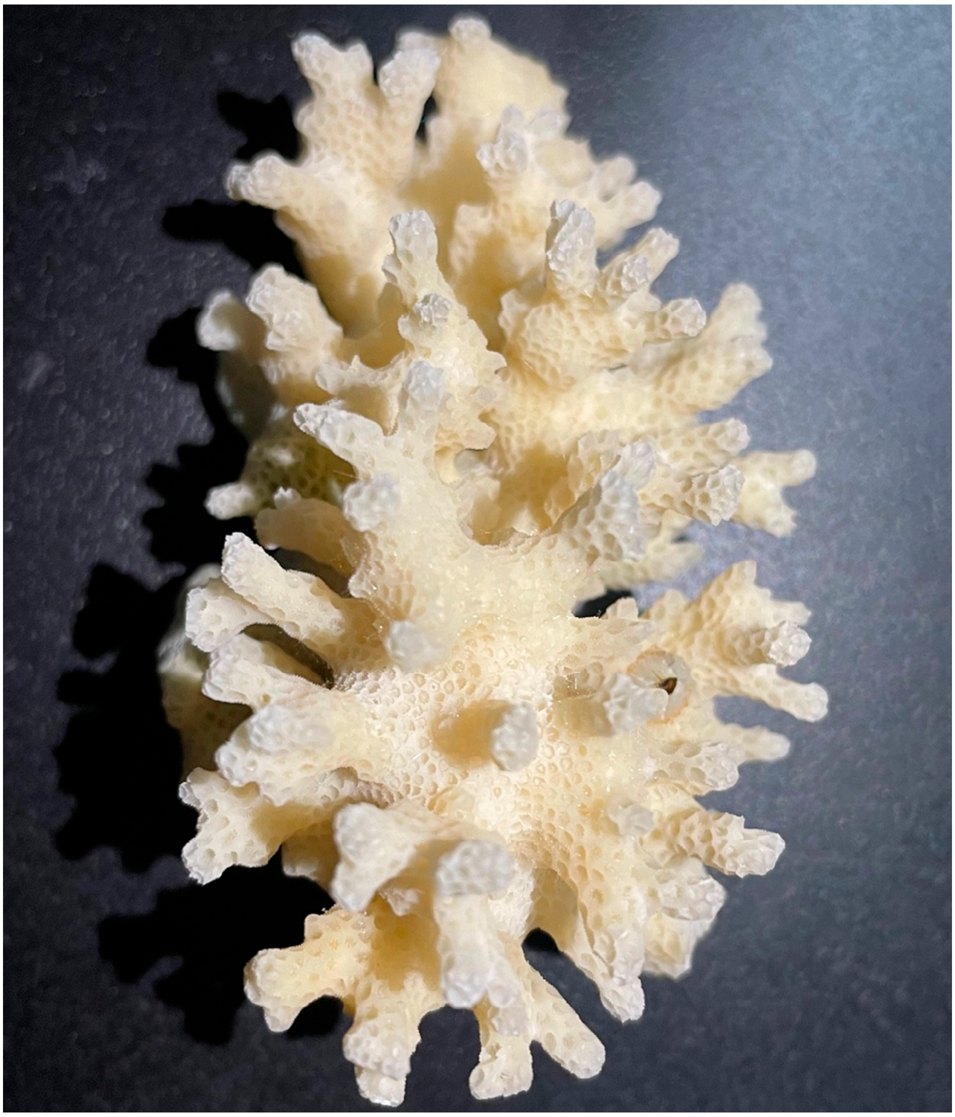

Figure S1. Coral skeleton removed tissue.

Figure. S2

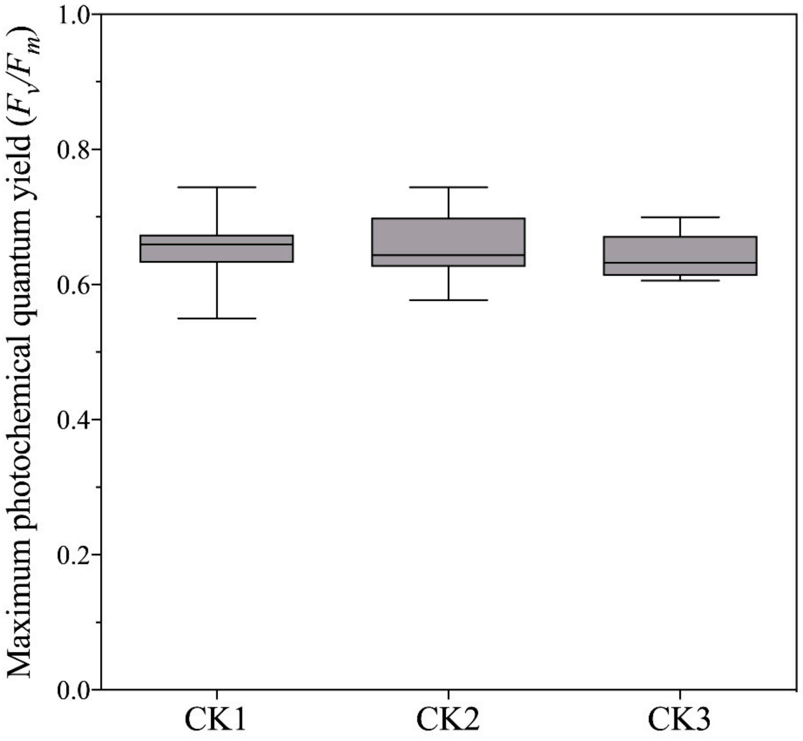

Figure. S2 Determination of the maximum photochemical quantum yield (F_v/F_m) of *Pocillopora* sp. in the control tank. The coordinates 26C and 26R represent the 26 °C control and 26 °C recovery, respectively. Coral nubbins (n=3) were collected for F_v/F_m determination. Each sample was randomly measured eight times. The data were subjected to nonparametric Kruskal-Wallis tests. There was no significant difference between each group.

Figure. S3

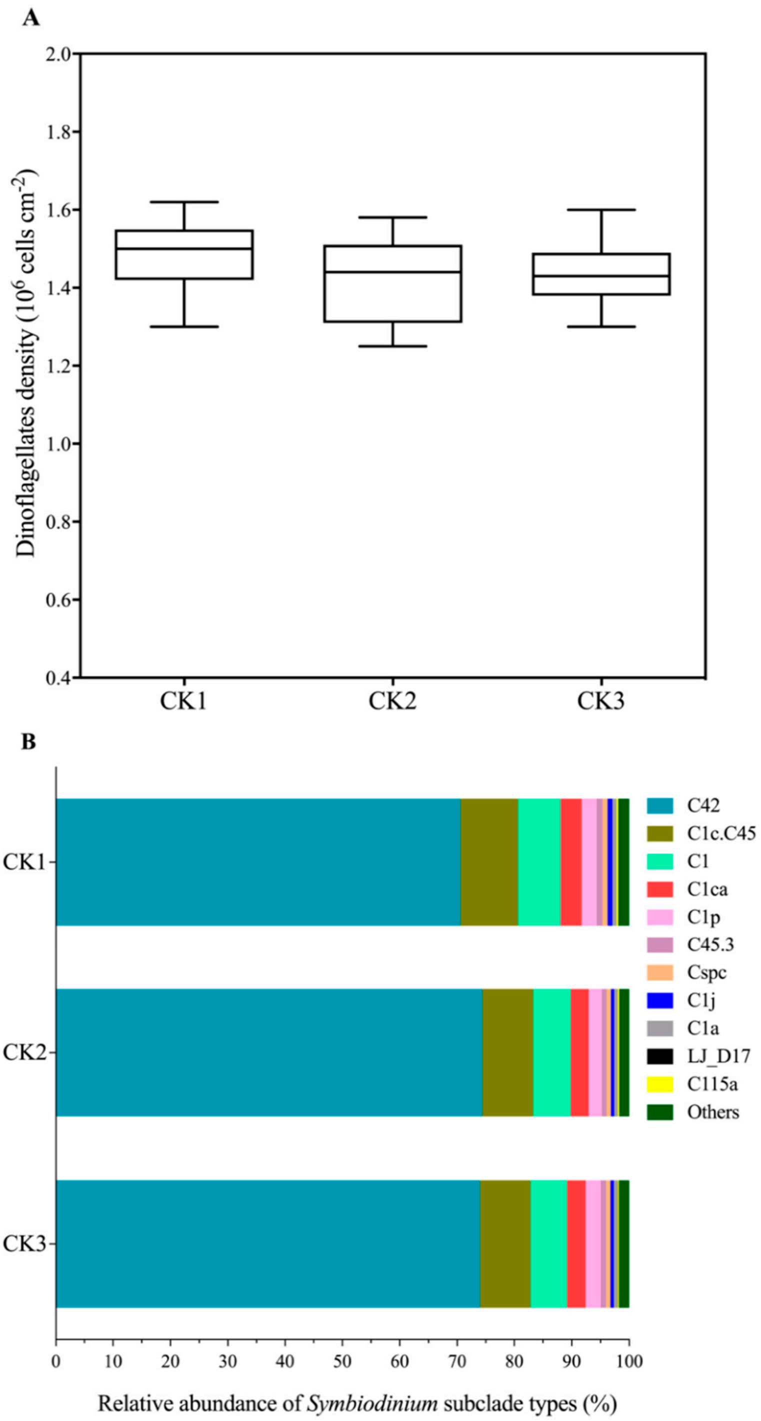

Figure. S3 Symbiodiniaceae density (A) and ITS2 sequence-based subclade composition (B) of *Pocillopora* sp. in the control tank. The data in (A) are the means ± SEs. "Others" (B) represent Symbiodiniaceae subclades whose members have a relative abundance of less than 0.01%. Coral nubbins (n=2) were collected in each stage to measure Symbiodiniaceae density and extract metagenomic DNA for detecting Symbiodiniaceae composition. Three replicates of Symbiodiniaceae density data were obtained for each sample. The data were subjected to nonparametric Kruskal-Wallis tests. There was no significant difference between each group.

Figure. S4

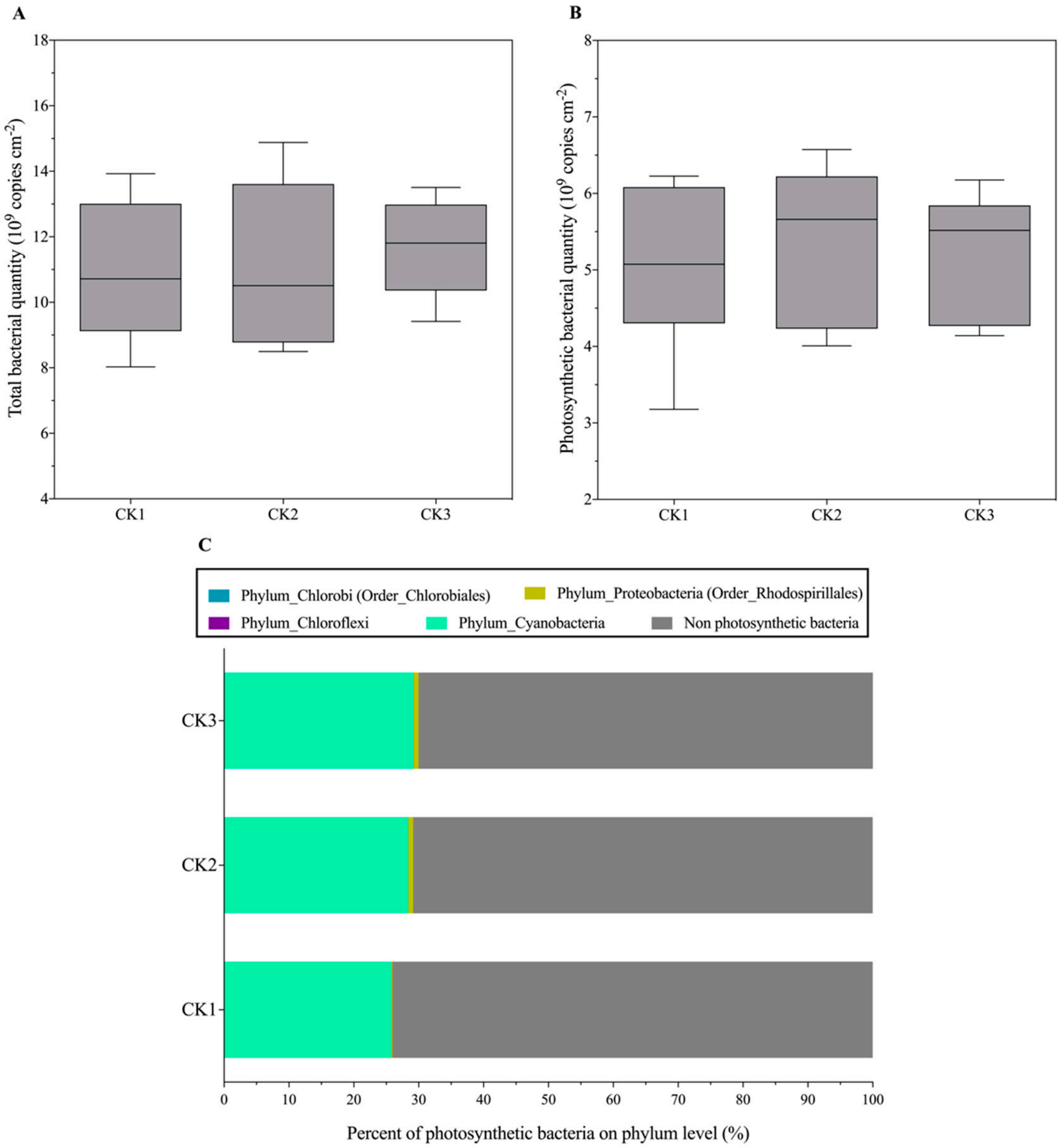

Figure. S4 Bacterial community composition and cells density in the coral *Pocillopora* sp. in the control tank. (A) and (B) show the absolute numbers of total bacteria and photosynthetic bacteria, respectively, in coral unit surface area (cm^{-2}). (C) shows the relative abundance of photosynthetic bacteria at the phylum level. The data in (B) and (C) are the means ± SEs. Coral nubbins (n=2) were collected in each stage for bacterial composition and quantification determination. Six replicates of the bacterial quantification data were obtained for each sample. The data were subjected to nonparametric Kruskal-Wallis tests. There was no significant difference between each group.

Figure. S5

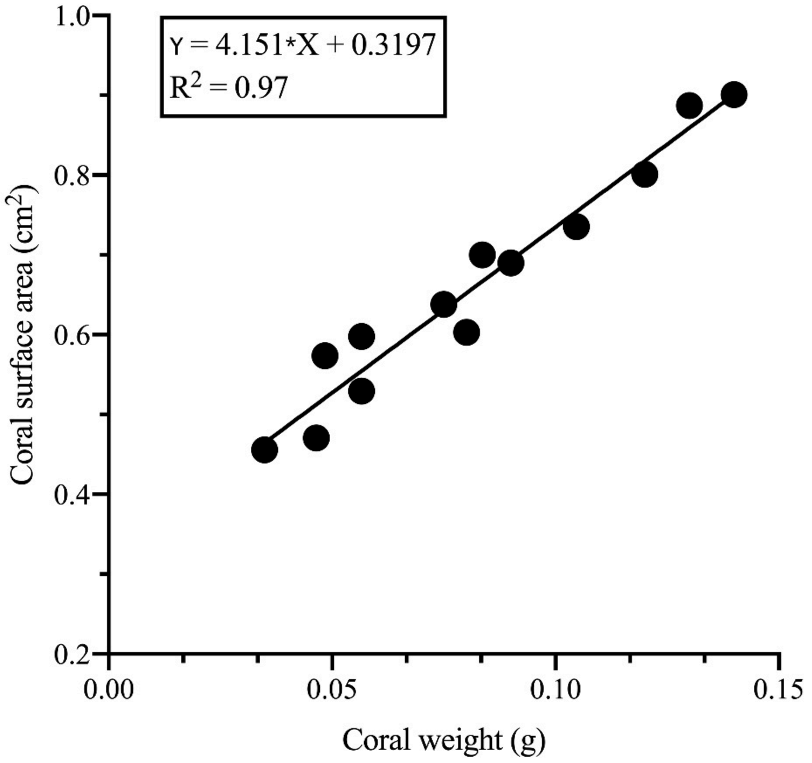

Figure. S5 Standard curve was constructed by the relationship between coral weight and surface area.
